# Use of titanium dioxide nanoparticles biosynthesized by *Bacillus mycoides* in quantum dot sensitized solar cells

**DOI:** 10.1186/s12934-014-0090-7

**Published:** 2014-07-16

**Authors:** Nicolás Alexis Órdenes-Aenishanslins, Luis Alberto Saona, Vicente María Durán-Toro, Juan Pablo Monrás, Denisse Margarita Bravo, José Manuel Pérez-Donoso

**Affiliations:** 1 Universidad Andres Bello, Bionanotechnology and Microbiology Lab, Center for Bioinformatics and Integrative Biology (CBIB), Facultad de Ciencias Biologicas, Republica # 239, Santiago, Chile; 2 Universidad de Chile, Facultad de Ciencias Químicas y Farmacéuticas, Sergio Livingstone Pohlhammer 1007, Santiago, Chile; 3 Universidad de Santiago de Chile, Facultad de Química y Biología, Alameda 3363, Santiago, Chile; 4 Universidad de Chile, Laboratorio de Microbiología Oral, Facultad de Odontologia, Sergio Livingstone Pohlhammer 943, Santiago, Chile

**Keywords:** Titanium dioxide nanoparticles, QDSSC, Phototoxicity, Nanoparticle biosynthesis

## Abstract

**Background:**

One of the major challenges of nanotechnology during the last decade has been the development of new procedures to synthesize nanoparticles. In this context, biosynthetic methods have taken hold since they are simple, safe and eco-friendly.

**Results:**

In this study, we report the biosynthesis of TiO_2_ nanoparticles by an environmental isolate of *Bacillus mycoides*, a poorly described Gram-positive bacterium able to form colonies with novel morphologies. This isolate was able to produce TiO_2_ nanoparticles at 37°C in the presence of titanyl hydroxide. Biosynthesized nanoparticles have anatase polymorphic structure, spherical morphology, polydisperse size (40–60 nm) and an organic shell as determined by UV–vis spectroscopy, TEM, DLS and FTIR, respectively. Also, conversely to chemically produced nanoparticles, biosynthesized TiO_2_ do not display phototoxicity. In order to design less expensive and greener solar cells, biosynthesized nanoparticles were evaluated in Quantum Dot Sensitized Solar Cells (QDSSCs) and compared with chemically produced TiO_2_ nanoparticles. Solar cell parameters such as short circuit current density (*I*_*SC*_) and open circuit voltage (*V*_*OC*_) revealed that biosynthesized TiO_2_ nanoparticles can mobilize electrons in QDSSCs similarly than chemically produced TiO_2_.

**Conclusions:**

Our results indicate that bacterial extracellular production of TiO_2_ nanoparticles at low temperatures represents a novel alternative for the construction of green solar cells.

## Introduction

The rapid advance of nanotechnology and the increasing number of applications involving nanomaterials have prompted the interest in developing simple and environmentally friendly protocols for nanoparticle synthesis.

To date, titanium nanoparticles (NPs) are one of the most required nanomaterials because of its use on different technological applications. Nanoparticulated titanium dioxide is a highly valuable material since it is used as photocatalyst degrading organic molecules in water treatment [[1]], white pigment in paint manufacturing, additive in food and personal care products [[2]], and composite films in biomedical sciences [[3]], among many other applications.

Since TiO_2_ nanoparticles can conduct electrons as a wide-band gap semiconductor, its use as photoanode in the manufacture of Dye- and Quantum Dots- Sensitized Solar Cells (DSSCs and QDSSCs, respectively) has gained importance during the last decade [[4],[5]]. This application has become more attractive during the last years due to the global need for replacing fossil fuels for energy generation. Accordingly, the research and development of non-conventional renewable energies (NCRE), particularly solar energy, which arises as a sustainable and abundant alternative to meet the high world energy demand, has been strongly stimulated [[6]].

Current methods to produce TiO_2_ nanoparticles involve different chemical procedures such as sol–gel [[7]], hydrothermal [[8]] and solvothermal [[9]], among others. All these methods involve high temperatures (>200°C) and in some cases elevated pressures, both conditions affecting the safety and costs of the process.

During the last years, NPs biosynthesis methods involving microorganisms [[10],[11]] or plant extracts [[12]], as well as eco-friendly chemical procedures involving low toxicity reagents and mild conditions of temperature and pressure, have taken hold [[13]]. This has allowed the development of nanoparticles displaying novel properties such as composition, size and biocompatibility [[14]–[20]]. Biological protocols for the synthesis of TiO_2_ nanoparticles have been developed, and the use of bacteria [[21]], yeasts [[22]], fungi [[23]] and plant extracts [[24]] have been recently reported (Table 1). Most biosynthetic procedures involve the use of titanyl hydroxide as precursor, and generate NPs with similar properties to those obtained by chemical procedures, such as size distribution and anatase/rutile crystal structure. In addition, most applications of biosynthesized TiO_2_ nanoparticles tested to date are based on their toxic properties against pathogens such as bacteria [[23],[25]] and mites [[26]]. Furthermore, *in vivo* and *in vitro* biocompatibility studies [[27]] have been carried out and their biocidal/photocatalytic activity on aquatic biofilms has been evaluated [[28]] (Table 1).

**Table 1:** **Biosynthesis of TiO**_**2**_**nanoparticles reported to date**

**Organism used in the biosynthesis**	**Gram**	**Precursor**	**Synthesis temperature**	**Particle size**	**Crystal structure (dominant)**	**Application**	**Reference**
***Saccharomyces cerevisiae***	Not applicable	Titanyl hydroxide	60°C	13 nm (TEM)	Anatase and Rutile	-	[[22]]
		TiO(OH)_2_		18 nm (XRD)			
***Lactobacillus*** **sp.**	(+)	Titanyl hydroxide	60°C	25 nm (TEM)	Anatase and Rutile	-	[[22]]
		TiO(OH)_2_		30 nm (XRD)			
***Bacillus subtilis***	(+)	Titanyl hydroxide	60°C	66-77 nm (SEM)	Anatase	-	[[21]]
		TiO(OH)_2_					
**Leaves extract of** ***Nyctanthes arbor-tristis***	Not applicable	Titanium tetraisopropoxide	1. 50°C	100 nm (XRD)	Not shown	-	[[29]]
		Ti(OCH(CH_3_)_2_)_4_	2. 500°C (calcined)	100–150 nm (SEM)			
**Aqueous extract of** ***Jatropha curcas L.*** **latex**	Not applicable	Titanyl hydroxide	50°C	25-100 nm	Anatase	-	[[30]]
		TiO(OH)_2_					
***Aspergillus flavus***	Not applicable	Titanium dioxide	37°C	62-74 nm	Anatase and Rutile	Against pathogenic bacteria	[[23]]
		TiO_2_					
**Leaf aqueous extract of** ***Eclipta prostrata***	Not applicable	Titanyl hydroxide	Room Temperature	49.5 nm	Rutile	-	[[24]]
		TiO(OH)_2_		(36–68 nm)			
***Annona squamosa*** **peel extract**	Not applicable	Titanyl hydroxide	60°C (Optimal)	26 nm (XRD)	Rutile	-	[[31]]
		TiO(OH)_2_		23 nm (TEM)			
***Bacillus subtilis***	(+)	Potassium hexafluorotitanate	1. Not shown	10-30 nm	Anatase	Photocatalytic activity on aquatic biofilm	[[28]]
		K_2_TiF_6_	2. 500°C (to crystallize particles)				
**Leaves extract of** ***Catharanthus roseus***	Not applicable	Titanium dioxide	50°C	25-110 nm (SEM)	Anatase and Rutile	Antiparasitic activity	[[32]]
		TiO_2_ (powder)		65 nm (XRD)			
**Flower aqueous extract of** ***Calotropis gigantea***	Not applicable	Titanyl hydroxide	90°C	160-220 nm (SEM)	Not shown	Acaricidal activity	[[26]]
		TiO(OH)_2_		10.52 nm (XRD)			
***Planomicrobium*** **sp.**	(+)	Titanium dioxide	50°C	100-500 nm (SEM)	Not shown	Antibacterial and antifungal activity	[[33]]
		TiO_2_		8.89 nm (XRD)			
***Propionibacterium jensenii***	(+)	Titanyl hydroxide	1. 60°C	65 nm (XRD)	Anatase	Preparation of collagen-TiO_2_ wound dressing	[[27]]
		TiO(OH)_2_	2. 300°C (annealed)	10–80 nm (FE-SEM)			
***Aeromonas hydrophila***	(−)	Titanyl hydroxide	30°C	40.5 nm (XRD)	Rutile	Antibacterial activity	[[25]]
		TiO(OH)_2_		28–54 nm (SEM)			
**Leaves extract of** ***Solanum trilobatum***	Not applicable	Titanyl hydroxide	Room Temperature	70 nm (SEM)	Rutile	Antiparasitic activity	[[34]]
		TiO(OH)_2_					

No reports regarding the application of biosynthesized titanium dioxide nanoparticles in energy devices have been published to date (Table 1). The use of other biologically produced materials in sensitized solar cells has been recently reported, however these reports focused on using plant pigments or channel proteins in the photon harvest process [[35],[36]].

The present work reports for the first time the use of biosynthesized titanium dioxide nanoparticles by *B. mycoides,* as semiconductors in the manufacture of photoanodes for QDSSCs. Along with introducing a new method for the synthesis of TiO_2_ nanoparticles, the present manuscript constitutes a first approach for using biosynthesized nanoparticles in solar cells and opens the interest in using other biosynthesized nanoparticles in energy devices as a way to develop greener photovoltaic technologies at low production costs.

## Results and discussion

### Environmental isolate of *Bacillus mycoides*

**Figure 1:**
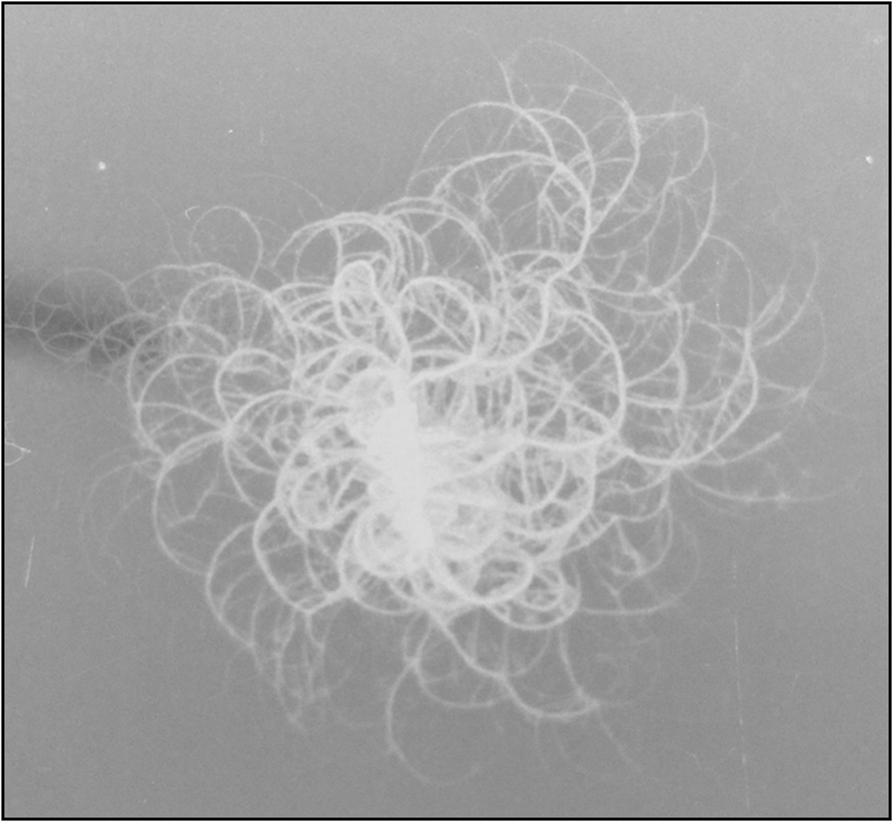
**Colony of** ***B. mycoides*** **isolate (SIN strain).**

A *B. mycoides* strain isolated from a soil sample obtained from a volcanic zone in Chile was used for biosynthesis of TiO_2_ NPs. *B. mycoides* is a member of the *Bacillus cereus* group of bacteria, a nonpathogenic soil and saprophyte Gram-positive bacilli. When grown on agar plates, this bacterium has the ability to form chains of cells that define macroscopic colonies with filaments projecting radially and curving in two possible orientations, clockwise (Dextral or DX strains) or counter-clockwise (Sinistral or SIN strains) [[37]]. The environmental isolate used in this work displays the classical colony morphology of *B. mycoides* with radial filaments in an anti-clockwise direction, indicative of a SIN strain (Figure 1).

Biosynthesis of TiO_2_ nanoparticles by the isolated strain of *B. mycoides* was carried out by exposing bacterial cultures to titanyl hydroxide at 37°C, the optimal growth temperature determined for this environmental isolate (not shown). Then, the temperature of the culture was diminished (20–25°C) to stop the reaction and a white precipitate was formed, indicative of TiO_2_ nanoparticles synthesis. The precipitate was purified from the culture, washed and resuspended in Mili-Q ultra pure water for subsequent studies.

To date, there are two studies reporting the use of the genus *Bacillus* for biosynthesis of TiO_2_ nanoparticles, however, these reports do not use titanyl hydroxide as precursor [[28]] and require high temperatures for the synthesis of NPs [[21]] (Table 1). Differences in the biosynthetic process mediated by *B. mycoides* suggest that different biomolecules could be involved in biosynthesis and that the produced NPs could display novel properties.

### Transmission electron microscopy (TEM)

**Figure 2:**
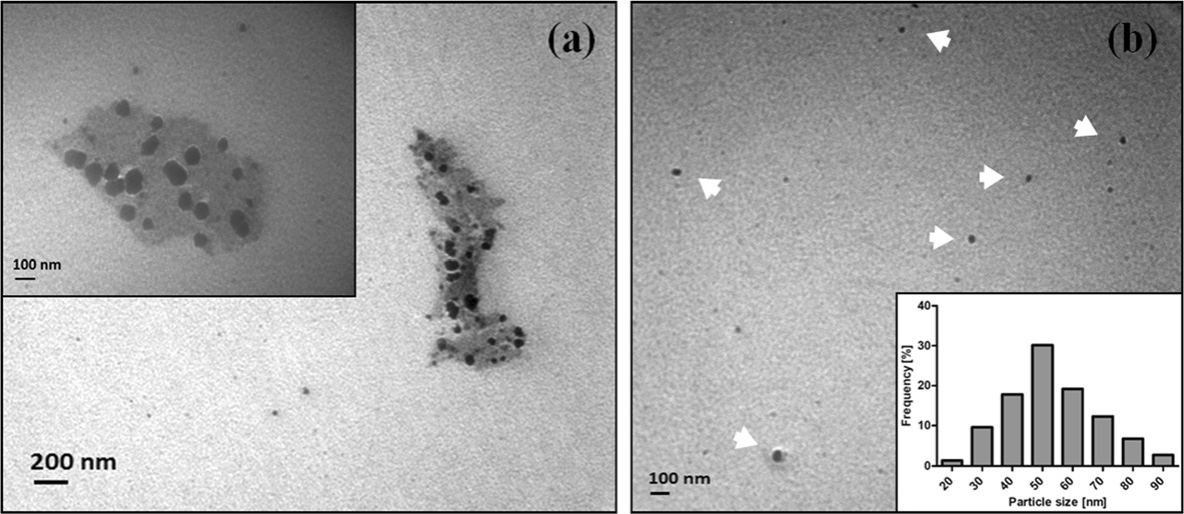
**Transmission electron microscopy of TiO**_**2**_**nanoparticles synthesized by*B. mycoides*.** Biosynthesized nanoparticles before **(a)** and after successive washings in water **(b)**. The inset in **(a)** shows the spherical morphology of TiO_2_ nanoparticles. The inset in **(b)** shows the size distribution histogram of nanoparticles. Arrow heads indicate individual NPs. Images were obtained with a 100,000× magnification.

A TEM analysis of the nanoparticles produced by *B. mycoides* was performed with the aim to determine their nanometric size and distribution. Biosynthesized TiO_2_ nanoparticles display a size between 40–60 nm and spherical morphology (Figure 2). The size distribution histogram indicates a high polydispersity of the sample; which is a common behavior of NPs produced by biosynthetic methods [[23],[24],[27]]. This result suggests that nanoparticles are produced by cells at different times after addition of titanyl hydroxide. TEM images of Figure 2a show nanoparticles coated by an organic envelope, probably corresponding to the extracellular matrix produced by *B. mycoides*. This matrix could participate in substrate biotransformation (titanyl hydroxide to titanium dioxide nanoparticles), or maybe could help stabilizing and/or capping the NPs. When this sample was further purified by successive washings steps using ultra pure water, individual nanoparticles were observed (Figure 2b).

### Fourier transform infrared spectroscopy (FTIR) analysis

**Figure 3:**
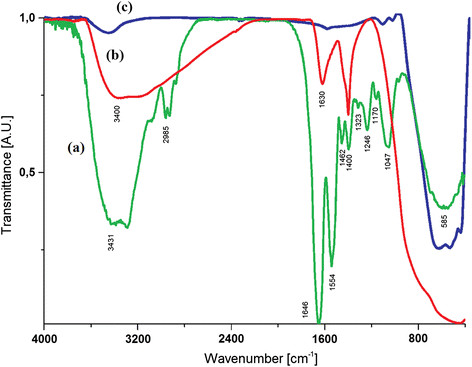
**FTIR spectra of TiO**_**2**_**nanoparticles biosynthesized by** ***B. mycoides*** **(a), the precursor TiO(OH)**_**2**_**(b), and TiO**_**2**_**nanoparticles produced by chemical synthesis (c).**

Figure 3 shows the FT-IR spectrum of titanyl hydroxide, biosynthesized titanium dioxide nanoparticles and chemically synthesized titanium dioxide nanoparticles. The FTIR spectrum of TiO(OH)_2_ shows the characteristic signals at 3400 cm^−1^ and 1630 cm^−1^ attributed to the presence of hydroxyl groups (Ti-OH) and water in their structure. The characteristic signal for TiO_2_ nanoparticles due to the vibration of Ti-O-Ti bond is observed at 450–700 cm^−1^ in both chemical and microbiological NPs. For biosynthesized TiO_2_ nanoparticles a broad band at 3431 cm^−1^ is observed. This signal corresponds to the O-H stretching due to the alcoholic group. The peaks around 2985 cm^−1^ are assigned to the symmetric stretch (C–H) of CH_2_ and CH_3_ groups of aliphatic chains. In 1646 cm^−1^, 1554 cm^−1^, 1462 cm^−1^ and 1400 cm^−1^ the characteristic signals of C = O and N-H vibrations due to the presence of amide and amine groups are shown. 1246 cm^−1^ corresponds to C-O stretch vibrations, possibly due to the presence of an alcohol or carboxylic acid group. The band at 1047 cm^−1^ corresponds to the C-N stretching vibrations of aliphatic amines. The peaks at 1554 cm^−1^ and 1400 cm^−1^ might also indicate the C = C ring stretching and bending vibration of CH_2_. All these signals can be attributed to the presence of biomolecules like peptides or carbohidrates bound to the TiO_2_ nanoparticles produced by *B. mycoides*. These biomolecules are part of the cell envelope of this bacterium and can provide support for the nucleation of the nanoparticles, and/or be involved in the biosynthesis process acting as stabilizing and capping agents.

### UV-visible spectroscopy and Tauc Plot analysis

In order to determine the UV-visible absorption spectrum of biosynthesized TiO_2_ nanoparticles, the product of biosynthesis was washed gently with ethanol and resuspended in ultrapure water to remove organic residues that might interfere with the measurement.

**Figure 4:**
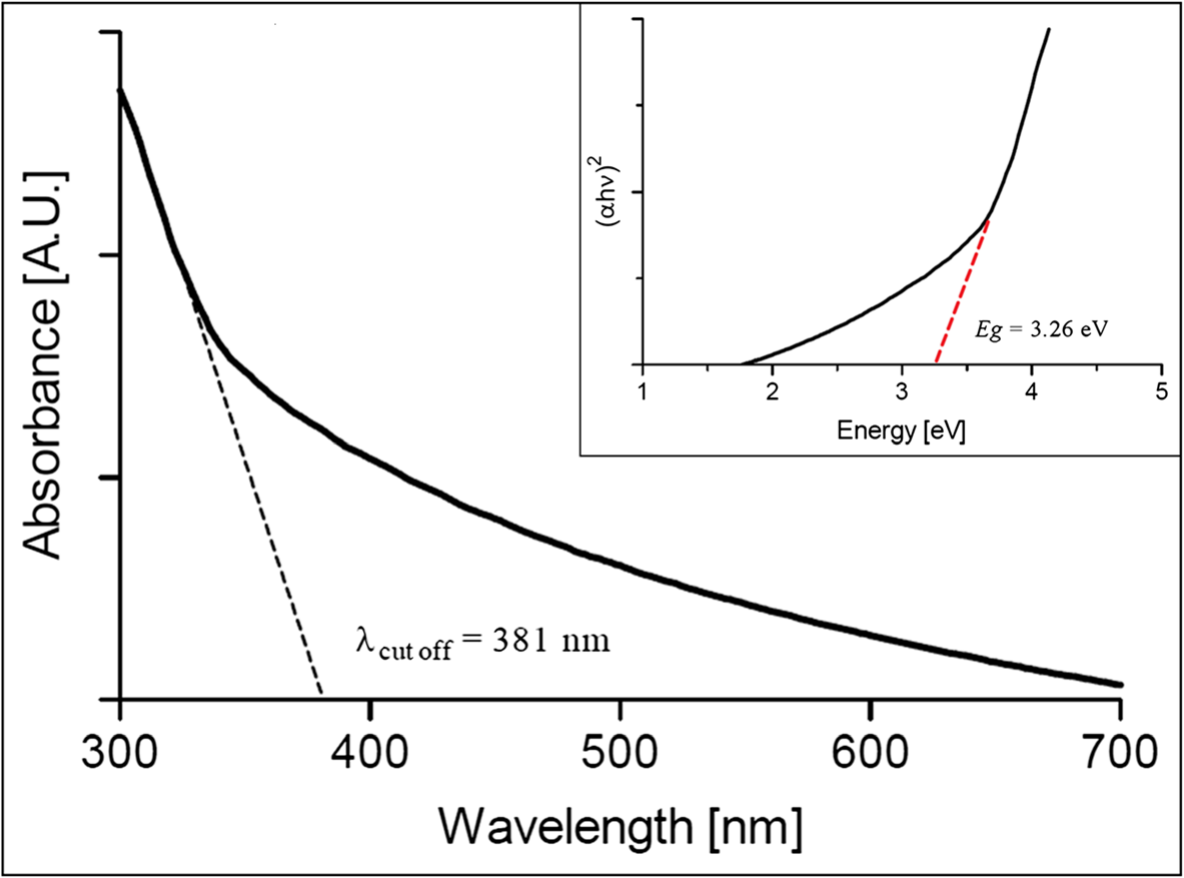
**UV-visible absorption spectrum of biosynthesized TiO**_**2**_**nanoparticles and Tauc plot used to determine its band gap (inset).**

The observed absorption spectrum coincides with those obtained with titanium dioxide nanoparticles produced by chemical methods, with a broad absorption band in the UV range and the cut off wavelength near 380 nm (Figure 4) [[27],[28],[38],[39]]. The obtained UV–vis spectra of biosynthesized TiO_2_ nanoparticles was used to determine the band gap (E_*bg*_) by the Tauc relation [[40]]. The method for determination of the E_*bg*_ value involves plotting (*αhν*)^2^ versus *hν*, where *α* is the absorption coefficient and *hν* is the energy of the incident photons. After making a linear fit to the curve, the value of the band gap is given by the value of the intercept of the line with the X-axis (*hν*-intercept) in this graph (Figure 4, inset) [[41]]. The band gap determined for biosynthesized TiO_2_ nanoparticles was 3.27 eV. This band gap value confirms that *B. mycoides* is producing titanium dioxide in anatase crystalline structure. Due to their wide band gap, TiO_2_ nanoparticles in anatase crystalline form are preferably used in sensitized solar cells [[42]]. This result suggests that biosynthesized nanoparticles are suitable semiconductor materials that can be used in QDSSCs.

### Antibacterial activity of biosynthesized TiO_2_ nanoparticles

Toxicity of TiO_2_ nanoparticles has become a relevant parameter since it can determine its use in different technological applications. Almost all publications related to biosynthesis of TiO_2_ NPs report the toxic and/or phototoxic effects of them (Table 1). Toxicity can decrease the number of technological applications in which TiO_2_ NPs can be used, but is the base of its use as antimicrobials and photo-reactive compounds. The use of nanoparticles in solar cells is not the exception and in addition to proper size, composition and semiconductor properties, increased biocompatibility will strongly favor their application in harvesting solar energy.

**Figure 5:**
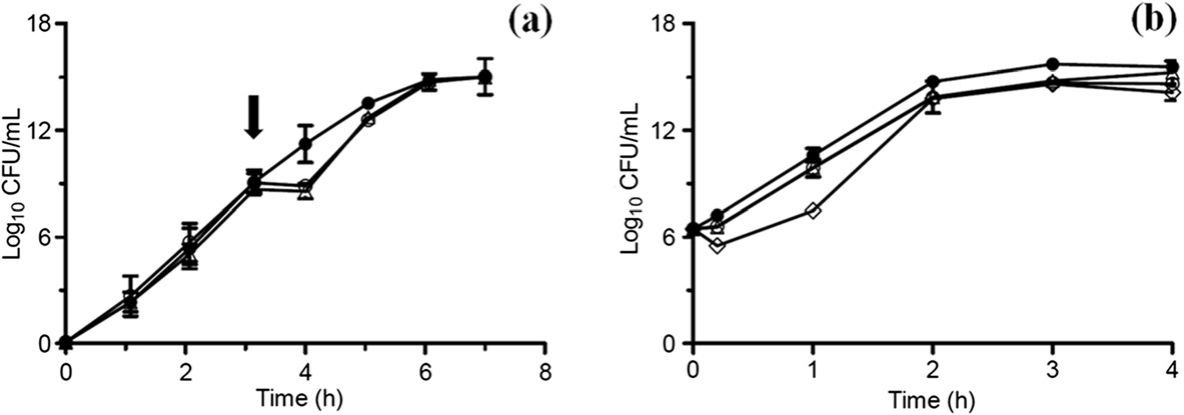
**Toxicity and phototoxicity evaluation of TiO**_**2**_**nanoparticles against*E. coli*. (a)***E. coli* CFUs after exposure to 200 μg/mL of chemical () and biological () TiO_2_ nanoparticles (control ). **(b)***E. coli* CFUs after treatment with 200 μg/mL of chemical () and biological () TiO_2_ nanoparticles exposed to UV light (control exposed to UV , control unexposed to UV ). The arrow in **a** indicates the time in which cells were treated with TiO_2_ nanoparticles.

As shown in Figure 5a, almost no toxicity was determined in chemical and biological nanoparticles. *E. coli* cultures were able to reestablish their growth after NPs exposure. To confirm our results, growth inhibition area assays were done, and no toxicity was determined for the biological and chemical nanoparticles evaluated (data not shown). Other studies have reported the biosynthesis of TiO_2_ nanoparticles displaying high toxicity for *E. coli,* with minimum inhibitory concentrations (MIC) near 20 and 40 μg/mL [[23],[25]]. In contrast, the nanoparticles produced by *B. mycoides* do not display any toxicity to *E. coli* at these concentrations.

Most of the damage that TiO_2_ nanoparticles produce on microorganisms has been associated to phototoxicity (Table 1). When photocatalytic activity against *E. coli* was evaluated, a small decrease in CFU was determined for chemically synthesized TiO_2_ (Figure 5b). This is due to lipid peroxidation on bacterial cell membranes produced by reactive oxygen species (OH•, O_2_^−^ and H_2_O_2_) generated when titanium dioxide NPs are irradiated with UV light [[43]]. When *E. coli* cultures were amended with biologically synthesized TiO_2_ NPs and exposed to UV-B light, no effect on cell viability was determined, indicating that biosynthesized NPs do not display phototoxicity under the evaluated conditions (Figure 5b). Based on these results we can speculate that the organic coating of nanoparticles produced by *B. mycoides* protects bacteria from the phototoxic damage by interacting with UV-produced radicals. In this context, additional purification steps decreasing the organic matter of NPs probably increase phototoxicity of biosynthesized TiO_2_ NPs.

### Characterization and I-V measurement of the quantum dot sensitized solar cells

Based on the favorable properties of biosynthesized TiO_2_ nanoparticles as wide-band gap semiconductors with low levels of toxicity, we decided to evaluate their use on solar cells.

**Figure 6:**
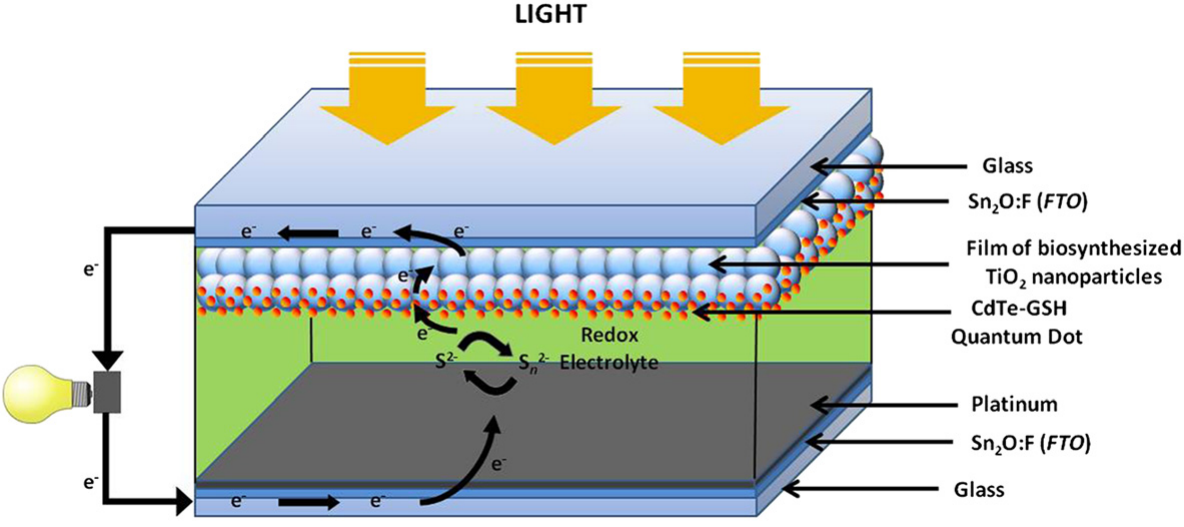
**Scheme of a quantum dot sensitized solar cell.** The film of biosynthesized TiO_2_ nanoparticles on conductive glass is sensitized with CdTe-GSH QDs forming the photoanode, while the cathode or counter electrode is platinum on FTO glass. The redox electrolyte fills the space between electrodes.

A schematic representation of the solar cell used to evaluate the biosynthesized TiO_2_ NPs is shown in Figure 6. When QDs absorb light, they inject electrons from their excited levels to the conduction band of the TiO_2_ nanoparticles film. The recirculation of the redox electrolyte in its oxidized-reduced state allows to recharge the electrons lost by oxidized QDs while serving as a pathway for electron transfer between the two electrodes [[5],[44]]. Thus, when light shines on the solar cell, the device directly converts sunlight into electricity and the current and voltage data can be recorded in an external circuit.

**Figure 7:**
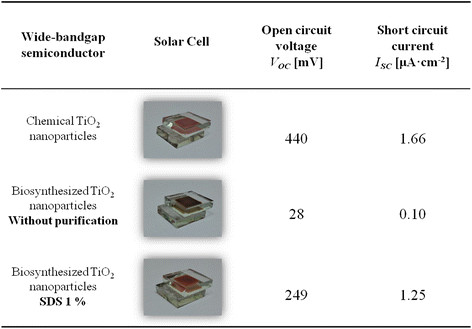
**I-V values of QDSSCs produced using biosynthesized TiO**_**2**_**NPs.**

The results of short circuit current density (*I*_*SC*_) and open circuit voltage (*V*_*OC*_) for the studied solar cells are summarized in Figure 7. The *I*_*SC*_ value corresponds to the maximum current (flow of electric charge) through the solar cell when the voltage in the device is zero. Moreover, the *V*_*OC*_ is the maximum voltage (electric potential difference) produced by the solar cell when the current flow is zero.

The control solar cells produced with chemical titanium dioxide nanoparticles display the highest values of voltage and current, confirming that the flow of electrons between the CdTe-GSH QDs and chemical TiO_2_ NPs works properly. Moreover, the QDSSC that uses biosynthesized TiO_2_ nanoparticles shows decreased values of *I*_*SC*_ and *V*_*OC*_, attributable to the presence of calcined organic matter on the surface of nanoparticles after sintering of the material; this calcined matter would work as insulation in electrical conduction. However, when the organic coating of TiO_2_ nanoparticles was removed, *V*_*OC*_ and *I*_*SC*_ values are significantly increased and the performance of the solar cell using biosynthesized TiO_2_ nanoparticles is similar to that observed in the control. The results obtained indicate that it is possible to use the TiO_2_ nanoparticles produced by *B. mycoides* in the development of greener solar cells.

## Conclusions

**Scheme 1:**
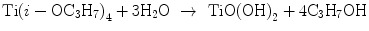
**Possible mechanism for biotransformation of titanyl hydroxide to titanium dioxide nanoparticles.** The dehydration reaction would be mediated by an acidic group present in an “unknown” component of the extracellular matrix of *B. mycoides* that could have a key role in biotransformation.

In this work the biosynthesis of titanium dioxide nanoparticles using an environmental isolate of *B. mycoides* was reported. Although the transformation of TiO(OH)_2_ to TiO_2_ normally occurs in a drying process or sol–gel combustion at temperatures between 150 and 400°C [[45]–[47]], in this paper we have shown that a *B. mycoides* isolate is able to biotransform this precursor into its nanostructured form at 37°C. A possible mechanism for TiO_2_ biosynthesis using titanyl hydroxide as precursor and a still unknown organic molecule from *B. mycoides* (X) is proposed in Scheme 1. Experiments to determine the identity of the organic molecule(s) involved in biosynthesis are under way in our laboratory.

TiO_2_ nanoparticles biosynthesized by *B. mycoides* exhibit low toxicity against *E. coli*, probably as consequence of the organic coating.

Biosynthesized nanoparticles are able to conduct electrons in QDSSC with values near those determined in a control solar cell produced with chemically synthesized TiO_2_ nanoparticles.

The main projection of this work is the use of these and other green nanoparticles in the sustainable manufacturing of solar cells to develop ecologically friendly and less expensive photovoltaic panels.

## Methods

### Synthesis of titanyl hydroxide precursor

Titanyl hydroxide was obtained by the hydrolysis of titanium isopropoxide [Ti(*i*-OPr)_4_] according to the following reaction [[46]]:

(1) $$\mathrm{Ti}{\left(i-{\mathrm{OC}}_{3}H_{7}\right)}_{4}+\;3H_{2}O\;\to \;\mathrm{TiO}{\left(\mathrm{OH}\right)}_{2}+\;4C_{3}H_{7}\mathrm{OH}$$

The reaction mixture was stirred for two hours under ice-cold condition until an opalescent suspension of TiO(OH)_2_ was obtained. The isopropyl alcohol (propan-2-ol) was removed by successive centrifugations and washings with Mili-Q ultrapure water. Finally, titanyl hydroxide was resuspended in water. The precursor concentration was obtained by determining the dry mass of a 1 mL solution, which was lyophilized (freeze-dried) for 24 h.

### Biosynthesis of TiO_2_ nanoparticles

A culture of 200 μL of *B. mycoides* grown overnight was used to inoculate 200 mL of LB medium (dilution 1:1000). This culture solution was grown for 12 h at 37°C with constant shaking (150 RPM). Then, 40 mL of a 25 mM titanyl hydroxide solution were added and the mixture incubated at 37°C for 24 h with constant shaking. After this time, the solution was incubated at room temperature for 8 h and the appearance of a white precipitate was indicative of the production of titanium dioxide nanoparticles. The precipitate was removed from the culture by centrifuging 15 min at 3820 × *g*. Finally, the biosynthesis product was washed and resuspended by successive centrifugations in Mili-Q ultra pure water.

### Characterization of TiO_2_ nanoparticles

Biosynthesized TiO_2_ nanoparticles were characterized by UV-visible spectroscopy using a Synergy™ H1 Microplate Reader (BioTek Instrument Inc.). Absorbance spectrum between 300–700 nm (2 nm resolution) was performed and used for band gap (E_*bg*_) determination using the Tauc relation [[41]].

For Transmission Electron Microscopy (TEM) studies, a suspension of TiO_2_ nanoparticles was deposited on a copper grid and examined using a Low Voltage Transmission Electron Microscope 5 (LVEM5) (Delong Instruments) operated at 5.1 kV. The size distribution histogram was performed using ImageJ software. Dinamic Light Scattering (DLS) was performed in a Zetasizer Nano ZS (Malvern Instrument Ltd.) equipment using the protocol previously described by our group [[10]].

For the Fourier Transform Infrared Spectroscopy (FT-IR) characterization samples were lyophilized (freeze-dried) for 24 h and the powder was mixed with KBr to form a thin pellet. FT-IR measurements were carried out using a Spectrum One FT-IR Spectrometer (Perkin Elmer Inc.) in the 400–4000 cm^−1^ range with a 4 cm^−1^ resolution.

### Antibacterial activity of TiO_2_ nanoparticles

The antibacterial activity of biosynthesized TiO_2_ nanoparticles was evaluated against *E. coli* (BW25113). Bacterial cultures were grown in LB medium at 37°C with constant shaking (150 RPM). After 3 h incubation (OD_600_ = 0.3) cultures were amended with 200 μg/mL of chemically (TiO_2_ nanopowder from Sigma-Aldrich, ~21 nm particle size) or biologically synthesized TiO_2_ nanoparticles. The photocatalytic effect of nanoparticles was evaluated using the same concentrations indicated above, but irradiating the culture with UV-B light for 2 min in the presence of the nanoparticles (OD_600_ = 0.3).

The effect of TiO_2_ nanoparticles on bacterial growth was evaluated by determining the number of colony forming units (CFU) over time. Culture aliquots were taken every hour and diluted to obtain 10^−1^ to 10^−7^ serial dilutions. 5 μL of every dilution were plated on LB agar, and incubated at 37°C for 12 h. After this time, CFU were determined.

### Fabrication and characterization of quantum dot sensitized solar cells

QDSSCs were produced following the protocols described by Bang *et al.* [[48]], Giménez *et al.* [[49]] and Pan *et al.* [[50]], with some modifications. To fabricate the electrodes of QDSSCs, 10 × 10 × 2 mm size fluorine doped tin oxide coated glass (FTO glass) TEC15, with a surface resistivity of 13 [Ω/sq] and 85% transmittance was used. Conductive glasses were cleaned by successive sonication in absolute ethanol and deionized water for approximately 10 min to remove organic contaminants. The anode was prepared using a suspension of biosynthesized TiO_2_ nanoparticles that was deposited on the glass through spin-coating at 2000 rpm for 10 sec.

To prepare a uniform titanium dioxide film that facilitates electron transfer process in QDSSCs, it is important to remove the organic coating on the surface of biosynthesized TiO_2_ nanoparticles. For this reason, an additional purification step was performed. Nanoparticles were treated with 1% sodium dodecyl sulfate (SDS) and the solution was sonicated gently for a few seconds to allow disaggregation of the nanoparticles. Then, the suspension of TiO_2_ nanoparticles was recovered by centrifugation, washed and resuspended in Mili-Q ultra pure water. Titanium(IV) oxide nanopowder from Sigma-Aldrich and biosynthesized TiO_2_ nanoparticles were used to manufacture the photoanodes of QDSSCs. The electrodes (TiO_2_ films) underwent a sintering process at 450°C for 30 min. Sensitization of TiO_2_ film was performed by direct adsorption of CdTe-GSH quantum dots (QDs) [[51]]. The active area of the cells was 0.16 cm^2^. Moreover, the cathode or counter electrode was prepared from a solution of H_2_PtCl_6_ · 6H_2_O in isopropanol. 10 μL of the solution were dispensed on a FTO coated glass by spin-coating and heated 20 min at 400°C.

Then, the photoanode and the counter electrode were assembled leaving 127 μm space between them. Before sealing the cell, a drop of electrolyte was added. The electrolyte solution used was sulfide/polysulfide (S^2−^/S_*n*_^2−^) prepared from Na_2_S (1.0 M), S (0.1 M) and NaOH (0.1 M) in Mili-Q ultrapure water. Characterization of solar cells was performed under constant conditions of temperature and irradiance at a one sun intensity as the light source (~100 mW · cm^−2^ and AM1.5).

## Competing interests

The authors declare that they have no competing interests.

## Authors’ contributions

NOA participates in the experimental design, carried out the precursor synthesis, microbiological work, built and characterized the solar cell, and drafted the manuscript. LSA isolated the bacteria and participated in the design of the experiments. JPM designed the experiments and purified the nanoparticles. VDT carried out bacterial toxicity experiments of nanoparticles. DB drafted the manuscript. JMP conceived the study, participated in the experimental design and coordination and writing of the manuscript. All authors have read and approved the final manuscript.
